# Experience of linking to the NHS diabetic eye screening programme records in the ASCEND-eye randomized trial and recommendations for improvement

**DOI:** 10.1016/j.conctc.2025.101474

**Published:** 2025-03-28

**Authors:** Emily Sammons, Louise Bowman, Marion Mafham, Jane Armitage

**Affiliations:** a Clinical Trial Service Unit & Epidemiological Studies Unit (CTSU), University of Oxford, Nuffield Department of Population Health, Richard Doll Building, Old Road Campus, Roosevelt Drive, Oxford, OX3 7LF, UK; b Medicine and Clinical Trials, CTSU, University of Oxford, Nuffield Department of Population Health, Richard Doll Building, Old Road Campus, Roosevelt Drive, Oxford, OX3 7LF, UK; c Heart Studies Group, CTSU, University of Oxford, Nuffield Department of Population Health, Richard Doll Building, Old Road Campus, Roosevelt Drive, Oxford, OX3 7LF, UK; d Health Data Research UK (HDR UK), London, UK; e Clinical Trials and Epidemiology, CTSU, University of Oxford, Nuffield Department of Population Health, Richard Doll Building, Old Road Campus, Roosevelt Drive, Oxford, OX3 7LF, UK

**Keywords:** Aspirin, Omega-3 fatty acids, Randomized controlled trial, NHS diabetic eye screening programme

## Abstract

**Background:**

The ASCEND-Eye sub-study of the large, double-blind, 2x2 factorial design, placebo-controlled ASCEND trial compared the effects of aspirin and, separately, omega-3 fatty acids on diabetic retinopathy outcomes derived from NHS Diabetic Eye Screening Programmes (DESP) in England and Wales, in adults aged 40 years or older with diabetes and no pre-existing atherosclerotic cardiovascular disease. ASCEND-Eye was unprecedented in what it set out to achieve; no previous studies had successfully obtained linked DESP data for research purposes on a national scale in England and Wales before.

**Objective:**

To describe our experience of linking DESP records to help other researchers wishing to use them. We explain the application process, lead times and resources required, and how these data were governed.

**Results:**

The process of gaining regulatory and ethics committee approval for ASCEND-Eye through to data acquisition took four years. Several challenges were encountered, including a lack of documentation defining the governance of the NHS screening service, the absence of a single central data repository, the inherent complexity of liaising with multiple data controllers, and a lack of responsiveness to invitations to collaborate by nearly half of the DESPs in England.

**Conclusion:**

Routinely collected healthcare data is a valuable source of outcome measure information in clinical trials. However, researchers frequently face barriers to accessing these datasets despite having written informed consent from trial participants to do so. We hope to encourage more NHS DESPs to take part in research.

## 1 Introduction

The UK has a wealth of high-quality registry data, which are a very valuable resource for recruiting and following participants in clinical trial [1,2] and observational research. [[3], [4], [5]]. The double-blind, 2x2 factorial design, randomized placebo-controlled ASCEND (A Study of Cardiovascular Events iN Diabetes) trial, conducted between 2005 and 2017, compared the effects of 100 mg aspirin and, separately, 1g omega-3 fatty acids daily for the primary prevention of serious cardiovascular events, in 15,480 UK adults, aged 40 years or older with diabetes. [[6], [7], [8], [9]]. The ASCEND-Eye sub-study linked 7360 of these participants to their NHS Diabetic Eye Screening Programme (DESP) records, with the primary aim of comparing the effect of both treatments on time to the first post-randomization occurrence of referable diabetic eye disease. We have previously published the sub-study's rationale, design and baseline characteristics [10] as well as the findings of a lack of any significant effect of these treatments on the primary outcome. [11,12]. In this paper, we describe our experience of applying for this linked screening data for trial participants in England and Wales and offer some suggestions to help others who may be embarking on similar studies.

## 2 Methods – The approval process

**Fig. 1:**
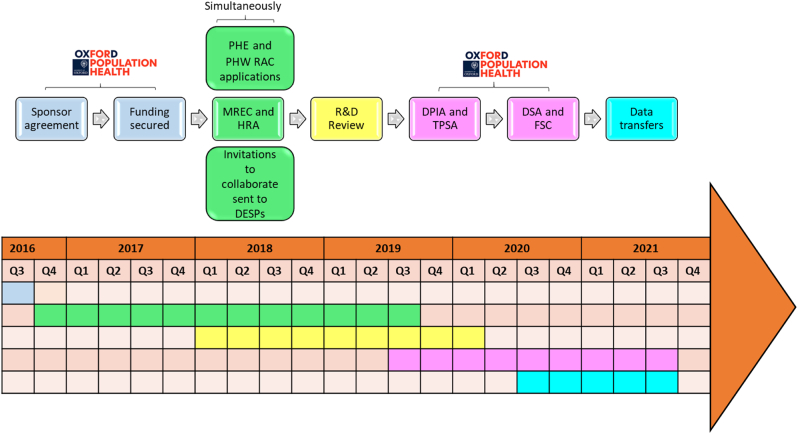
Schematic of the pre-requisites and approvals required to obtain the DESP linked data in ASCEND-Eye DESP = Diabetic Eye Screening Programme(s); DPIA = Data Protection Impact Assessment; DSA = Data Sharing Agreement; FSC = Financial Services Contract; HRA = Health Research Authority; MREC = Multi-centre Research Ethics Committee; PHE = Public Health England; PHW = Public Health Wales; RAC = Research Advisory Committee; R&D = Research and Development; TPSA = Third Party Security Assessment.

Fig. 1 summarises key steps in the governance processes required to deliver ASCEND-Eye within the research ethics framework of the NHS, the legal framework of the UK, and a few study-specific considerations. The following paragraphs describe each of these steps in more detail. While elements of our experience will be similar to those of other studies that are planning to use the NHS DESP data, for example, the requirement to obtain a well-defined sponsor, funding, regulatory and ethics committee approval, and the Diabetic Eye Screening Research Advisory Committee approval, there may also be additional requirements that are specific to individual trials. A summary of each application and its approval date is presented in Table S1 in the supplementary materials.

### 2.1 Sponsorship and funding

The University of Oxford sponsored ASCEND, meaning it took legal responsibility for ensuring that the trial was conducted safely, ethically and according to regulatory standards. The Clinical Trial Service Unit & Epidemiological Studies Unit (CTSU) in the Nuffield Department of Population Health (NDPH), also at the University of Oxford, was responsible for administering and coordinating the main trial and its sub-studies. With a clearly defined sponsor in place, it was then necessary to demonstrate that funds were available to deliver ASCEND-Eye. Grants to the University of Oxford were secured from the Oxford 10.13039/501100000274British Heart Foundation Centre of Research Excellence to cover the linkage fees. The department also received support from the 10.13039/501100000265UK Medical Research Council (which funded the 10.13039/501100000265MRC Population 10.13039/100005622Health Research Unit in a strategic partnership with the 10.13039/501100000769University of Oxford until March 2024), the 10.13039/501100000274British Heart Foundation and 10.13039/501100000289Cancer Research UK to cover staff costs. Funders had no role in collecting, analysing, or interpreting data or preparing any of the manuscripts for ASCEND-Eye.

### 2.2 Study-specific considerations

Longitudinal information about ASCEND participants' diabetic eye disease was obtained from electronic linkage to NHS DESP records in England and Wales. Due to the complexity of obtaining separate regulatory approvals in Scotland and Northern Ireland, a pragmatic decision was made to exclude the small number of participants who resided in these countries at recruitment (n = 506). [10]. Data linkage involves joining two or more datasets by a common matching variable, or ‘identifier’. The UK General Data Protection Regulation (GDPR), UK Medicines for Human Use (Clinical Trials) Regulations, and regulatory authorities require accuracy of these linkages to preserve the privacy of study participants (otherwise known as the ‘Data minimization principle’). When ASCEND-Eye was being planned, England had no single central repository of DESP data. Records were either jointly controlled by local service providers (NHS Trusts) and Public Health England (PHE, now the UK Health Security Agency; 40 DESPs) or solely controlled by one of two commercial providers of the screening software, Northgate Public Services (7 DESPs; which became NEC Software Solutions UK Ltd in July 2021) and InHealth Intelligence Ltd (11 DESPs). Participants were not asked which DESP they attended during ASCEND and they were also widely distributed throughout the UK, reflecting the mail-based design of the trial. [6,7]. To reduce the risk of sharing participants' identifiable data with DESPs they did not attend, a two-stage process of linking participants to their screening service was used. First, participants were linked to every GP practice they had ever been registered with during ASCEND, based on information they provided at enrolment or from updated correspondence during the trial. Next, PHE separately provided information to link GP practice codes to their serving DESPs. Finally, all three pieces of information were connected: participants were linked to their GP practices and then to their DESP(s). By contrast, a single registry existed in Wales, allowing participants who resided in Wales to be linked directly to Diabetic Eye Screening Wales (DESW) programme records, which were solely controlled by Public Health Wales (PHW).

The organization of DESPs in both countries meant that the delivery of ASCEND-Eye required careful consideration of the data protection risks involved, as well as the coordination of separate approvals from multiple data controllers and processors. Other challenges of our study included the age of the ASCEND trial, which began its recruitment in 2005. This predates the current legal framework of GDPR and the contemporary Integrated Research Application System (IRAS). Whilst neither issue precluded the approval of ASCEND-Eye, both delayed the processing of our regulatory and ethics applications. Finally, the close proximity of ASCEND's recruitment to the initiation and expansion of the eye screening service in England and Wales between 2003 and 2008, and changes in the geographical boundaries served by some programmes during the trial affected the coverage of data we could access.

### 2.3 Regulatory and ethics committee approval

In September 2016, a proposal was submitted and approved by the University of Oxford's Clinical Trials and Research Governance Department (now called the Research Governance, Ethics & Assurance Department) to make additional assessments of eye health in ASCEND. They were asked to approve a sub-study protocol and participant-facing materials, including a Participant Information Leaflet and Privacy Notice, prepared in line with the shared Information Commissioner's Office and NHS Digital transparency checklists, following consultations with the University of Oxford's Information Security and Information Governance teams. After institutional approval was given, a substantial amendment application was submitted and subsequently approved by the Northwest-Haydock Multi-Centre Research Ethics Committee (MREC) in October 2016. Another substantial amendment was approved in December 2016, which sought approval to extend the main ASCEND trial ethics approval to 2037 to permit long-term follow-up of ASCEND participants and to allow for sub-studies, such as ASCEND-Eye. A third substantial amendment was approved in January 2019 to add DESP registries under the legal definition of a research “site” that would perform the data linkage. Although some NHS Trusts were previously defined as Participant Identification Centres (PICs) for ASCEND, others were not formally involved with the parent study. By changing their role to a research “site” or opening new locations for ASCEND-Eye, it was necessary to bring the project under the Health Research Authority's (HRA) approval. Along with the MREC documentation, this required a ‘Schedule of Events’ and a ‘Statement of Activities’. Both the MREC and 10.13039/100009021HRA applications require that the sponsor (or their delegate) have, at a minimum, the following:

1. Data-sharing agreements with each data controller;
2. Adequate Information Security certification (e.g. ISO27001);
3. Arrangements for the secure storage and handling of data by each data processor; and
4. A legal basis to access the data.

Concurrent with these activities, two applications were sent to the Diabetic Eye Screening Research Advisory Committees in PHE (now the Research and Innovation Development Advisory Committee for NHS England) and separately, PHW, who have been responsible for commissioning the NHS screening programmes in England and Wales, respectively, since 2013. Approval to use the screening data for research purposes, as opposed to service planning, was given by PHE in July 2018 and PHW in September 2019. Both committees required annual progress reports for the duration of the study.

Simultaneously with preparing for and seeking these central approvals, a letter endorsed by Professor Peter Scanlon, Clinical Director of the NHS Diabetic Eye Screening Programme in both nations, was sent to the Clinical Lead(s) and Programme Manager(s) of 58 DESPs in England and to DESW, to invite their collaboration on the project. Those who responded favourably were sent the relevant regulatory authorizations and given the option of allowing their software provider (Northgate or InHealth Intelligence) to do the linkage on their behalf. In response to the specific requirements of the Research and Development (R&D) department in one NHS Trust, a fourth substantial amendment was submitted and subsequently approved by the MREC in December 2019 to clarify the integration of ASCEND-Eye and its documentation into the ASCEND trial protocol.

### 2.4 Data Sharing Agreements and Financial Service Contracts

The working models used to obtain approval for the data linkage from DESPs who responded favourably differed depending on who controlled the data, as follows:

1. The R&D offices of Trust/PHE-controlled DESPs in England were sent the regulatory authorizations described earlier and asked to sign a Data Sharing Agreement (DSA). They were also required to provide ‘Confirmation of the Capacity and Capability’ to collaborate on the project in accordance with HRA requirements.
2. For the company-controlled DESPs, the sponsor required separate ‘Third Party Security Assessments’ to be completed to verify the data governance credentials of Northgate and InHealth Intelligence before the regulatory submission package was sent to the Data Protection Officer in each company. Each company was asked to sign bespoke DSAs, which clearly defined the data processor-controller relationships at each stage of the data handling. Finally, a separate Financial Service Contract was used to authorize payments to Northgate for their work on the project; InHealth Intelligence provided their collaboration for free.
3. A separate approval was needed from the Research and Evaluation Division of PHW to permit linkage to their single registry for participants who resided in Wales. Personnel from PHW who could write a script and interrogate their local database were identified, and a bespoke DSA was used to define the roles and responsibilities of those involved in this process.

Each DSA and the Financial Service Contract was prepared and revised by the University of Oxford's Legal Officers.

### 2.5 Data Protection Impact Assessment

A Data Protection Impact Assessment (DPIA) was prepared in conjunction with the University of Oxford's Information Security and Information Governance teams to identify and mitigate the risks involved with processing personal data by the third parties. This received institutional approval in October 2020.

### 2.6 Legal basis

The lawful basis for processing personal data in ASCEND-Eye was that it was research carried out in the public interest (Article 6(1)(e) GDPR). The additional condition met for the processing of the special category data was that it was necessary for the purposes of research (Article 9(2)(j) GDPR).

### 2.7 Linkage process

After contracts relevant to the three working models were fully executed, a minimal dataset, which included the participants' unique ASCEND Trial ID number and NHS number, was encrypted before being sent to a nominated person at the relevant Trust, software company or DESW. NHS numbers were chosen as the sole linkage identifier because they are a unique pseudonym that remains the same throughout an individual's lifetime and have previously been shown to be valid and complete for the majority of primary and secondary care records in England. [13]. After linkage, data in the format of a comma-separated value or xls file, were encrypted again before being returned. In England, all data transfers were between nhs.net email accounts; in Wales, a file-sharing portal of equivalent security was used. Passwords to decrypt each file were sent via a separate communication channel for both directions of data transfer.

### 2.8 Information security

Data were collected and stored by the coordinating centre. All data, totalling 20.9 megabytes, was retained in an area of the NDPH main server (called QNAP), which was kept secure behind a default deny-all firewall. Processing of all identifiable data took place within the QNAP area. De-identified data points were transferred to the ASCEND database, which is held outside the secure QNAP area. Access to the data was controlled by personal user accounts and permission controls and restricted to substantive employees of the study. This included the principal investigators and other senior members of the trial team, including an administrator, data analyst, and trial statistician, who were expected to comply with the provisions of the Information Security and Data Protection Policies at NDPH, which are based on standards set out in the following legislation:

• NHS Data Security and Protection Toolkit
• Data Protection Act 2018
• General Data Protection Regulation (GDPR)
• Computer Misuse Act 1990
• ISO 27001:2017
• The Regulation of Investigatory Powers Act 2000
• Freedom of Information Act 2000

The coordinating centre conducted all analyses, which are described in separate publications. [[10], [11], [12]].

## 3 Results

The inclusion of participants in the primary efficacy analyses of ASCEND-Eye has been described elsewhere. [10]. Briefly, 13,656 (88 % of those randomised in ASCEND) participants were eligible to be linked. However, identifiers were only sent to be linked for 8108 (52 %) who attended a collaborating DESP. Of those, 7535 (49 %) were successfully matched, and 7360 (48 %) had retinopathy screening records within the scheduled treatment period of ASCEND.

**Fig. 2:**
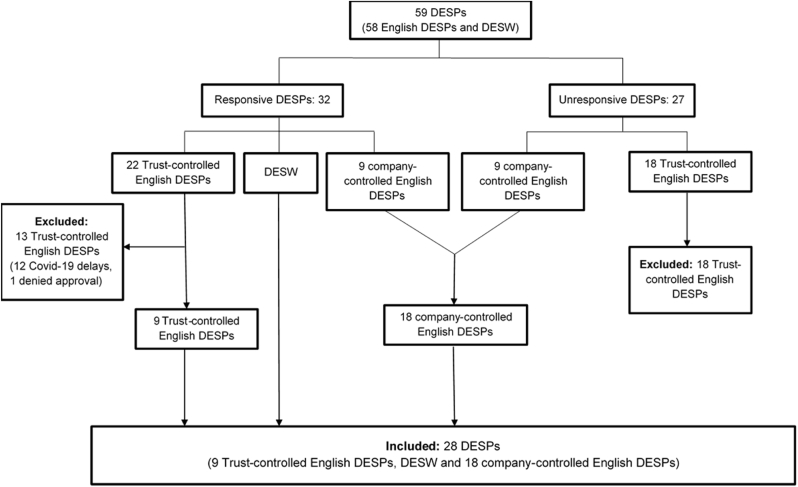
Consort diagram of DESP collaboration.

Fig. 2 is a consort diagram of the DESP collaboration in ASCEND-Eye. Table 1 summarises the number of participants thought to attend each DESP and their collaboration status in the study. Out of 59 programmes (58 English DESPs and DESW), 32 (54 %) responded favourably to the first invitation to collaborate or a second reminder, and 27 (46 %) were unresponsive. Those who responded included 22 Trust-controlled English DESPs, 9 company-controlled English DESPs, and DESW. Those who did not respond included 18 Trust-controlled English DESPs and 9 company-controlled English DESPs. Only Northgate and InHealth Intelligence needed to approve access to the data they controlled, but the Clinical Leads and Programme Managers of these 18 programmes (9 responsive and 9 unresponsive) were invited to collaborate as a courtesy. Approvals were obtained for 28 DESPs. This included 9 Trust-controlled English DESPs, all 18 company-controlled English DESPs and DESW. One R&D office acting on behalf of a Trust-controlled English DESP made the unilateral decision to deny approval for the study. The average time for R&D reviews in the nine English Trusts was 8.5 months (range of 1–18 months); PHW also took 8.5 months from application to approval. Separate approvals were obtained to link to the data controlled by Northgate (7 DESPs) and InHealth Intelligence (11 DESPs) by September 2021. The remaining 12 Trust-controlled English DESPs that expressed their willingness to collaborate suspended all non-coronavirus research at the start of the Covid-19 pandemic in March 2020, but this was nearly 16 months after they were first sent a submission package containing information about ASCEND-Eye and its regulatory and ethical approvals. For pragmatic reasons, a decision was made not to further pursue these 12 nor the 18 unresponsive Trust-controlled English DESPs. Therefore, overall, fewer than half of the 59 programmes contributed data to ASCEND-Eye (28 DESPs; 47 %). This was despite having the written informed consent from ASCEND participants and centralized approvals for the study. Fortunately, the involvement of 18 company-controlled DESPs, meant that a larger proportion of the randomised participants could be included.

**Table 1:** Number of Participants who attended each DESP by their Collaboration Status.

Collaborators				Non-Collaborators		
Trust-controlled DESPs	Northgate-controlled DESPs	Health Intelligence-controlled DESPs	Wales	Trust-controlled DESPs that were willing to collaborate prior to the Covid-19 pandemic	R&D approval denied	Unresponsive DESPs
Cheshire (203) East and North Hertfordshire (67) Greater Nottingham (369) Humber (370) North East London (41) North Nottinghamshire (260) North Yorkshire (320) Oxfordshire (275) South East Sussex (50)	Arden, Herefordshire and Worcestershire (273) Bath, Somerset, Swindon and Wiltshire (213) Central Mersey (25) North of Tyne and Gateshead (401) South West London (189) Surrey (469) West Riding and Craven (130)	Berkshire (136) Bristol and Weston (260) Devon (775) Dorset (117) East Anglia (460) Essex (360) Greater Manchester North (322) Greater Manchester South (128) Hampshire and Isle of Wight (761) Kent and Medway (318) North West London (180)	Diabetic Eye Screening Wales (853)	Birmingham, Solihull and Black Country (111) Brighton and Sussex (95) Derbyshire (747) Doncaster and Bassetlaw (76) Gloucestershire (184) Leicester, Leicestershire and Rutland (122) Norfolk and Norwich (176) Somerset (263) South East London (30) South Tees (148) Staffordshire (339) Shropshire (225)	Leeds and Mid Yorkshire (621)	Barnsley and Rotherham (38) Bedfordshire (44) Buckinghamshire (397) Cornwall (318) County Durham and Darlington (75) Cumbria (91) Lancashire (110) Lincolnshire (189) Liverpool (200) North Central London (37) North Mersey (10) North Tees (7) Northamptonshire (189) Sheffield (288) Sunderland and South Tyneside (146) West Hertfordshire (61) West Sussex (203) Wirral (8)
**Total number of programmes**						
**9**	**7**	**11**	**1**	**12**	**1**	**18**
**Total number of participants thought to have ever attended each DESP service**						
**1955**	**1700**	**3817**	**853**	**2516**	**621**	**2411**

Figures in parentheses represent the anticipated number of participants who were thought to have attended each DESP during the scheduled treatment period of ASCEND. Some participants were linked to more than one DESP due to changes in the geographical boundaries of each programme or because they moved their general practice. Therefore, the total number of participants across every DESP (n = 13,873) exceeds the total number eligible (n = 13,656) for the linkage exercise.

## 4 Discussion

ASCEND-Eye exemplifies the opportunity for maximizing the value of routinely collected healthcare data to support research when there is written informed consent from study participants. However, gaining regulatory and ethics approval for the DESP linkage was an iterative, time-consuming, and often demoralizing process. It necessitated a familiarity with complex legal terminology and governance structures and coordinating multiple applications to different authorities, each with specific requirements. Ideally, the study team would have understood the data governance of the national retinopathy screening service earlier in the project. However, this was hindered by a lack of documentation identifying who owned the data and needed to approve its use, the limitations on using the data and, therefore, the separate applications needed in addition to overarching authorizations from the MREC and HRA. The next challenge we faced was the innate complexity of liaising with multiple individuals in different agencies. Although similar information was included in each R&D application, all required additional forms, which differed in format and detail. Most then requested further information, assurances or alterations of these documents, the DSA, or both. Specific factors which delayed the processing of the submission package or caused confusion included the age of the original ASCEND registrations and the ongoing validity of participant consent, the frequent turnover of R&D staff, infrequent Research Advisory Committee meetings in PHE, fears of a legal misstep in the event of a data breach, and justifying the need for pseudonymized participant identifiers. In addition, we were required to adapt the HRA's ‘Schedule of Events’ and a ‘Statement of Activities,’ which are intended to be used in prospective randomized trials and were, therefore, largely irrelevant in the context of ASCEND-Eye.

Overall, it took over four years to obtain the final linked data set for ASCEND-Eye. The main difficulty associated with these delays was maintaining enthusiasm for the project with the funding bodies. Our recommendations from this experience include:

i) identify data-governance issues specific to the dataset being linked to at an early stage;
ii) involve institutional Information Governance, Finance and Legal teams from the outset of a project;
iii) request a single point of contact within Trust R&D teams; and
iv) ask data controllers to identify themselves in the subject line of each email, which helps to organize the correspondence.

At a higher level, regulatory application forms need to be tailored to the retrospective collection of data for linkage studies like ASCEND-Eye, and central authorizations need to replace lengthy governance reviews by individual Trust R&Ds. For example, data providers should not have to be defined as research sites, and the Confidential Advisory Group could advise on the validity of participant consent under common law. In addition, using the NHS Data Security and Protection Toolkit, an online self-assessment tool that enables organizations with access to NHS data to measure their information security, should be recognized by all NHS organizations.

In September 2021, it was announced that the eye screening service would be required to submit data to the National Diabetes Audit (NDA) each year, which should help to simplify future research applications. Despite its challenges, our study was unprecedented in what it set out to achieve. While NHS DESPs have been used by researchers before, previous work has tended to use anonymised cohorts from screening programmes in a single geographical location to inform service provision. [[14], [15], [16], [17]]. To the best of our knowledge, no prior studies have successfully obtained linked data for a clinical drug trial on a national scale in England and Wales before.

## CRediT authorship contribution statement

**Emily Sammons:** Writing – review & editing, Writing – original draft, Methodology, Formal analysis, Data curation, Conceptualization. **Louise Bowman:** Writing – review & editing, Supervision, Methodology. **Marion Mafham:** Writing – review & editing. **Jane Armitage:** Writing – review & editing, Supervision, Methodology, Conceptualization.

## Contributors and sources

JA and LB were co-principal Investigators of the ASCEND trial, and together with ES, they made substantial contributions to the design of the ASCEND-Eye study. ES obtained regulatory and information governance approvals, established collaborations with NHS Diabetic Eye Screening Programmes and their software providers, and wrote the manuscript we present. MM advised on broader aspects of the current regulatory environment for research. The corresponding author attests that all listed authors meet authorship criteria and that no others meeting the criteria have been omitted.

## Patient involvement

We did not seek input from participants during the preparation of this manuscript.

## Licence

For the purpose of open access, the authors have applied a Creative Commons Attribution (CC BY) license to any Author Accepted Manuscript version arising.

## Data availability statement

No additional data are available.

## Study registration

Eudract No. 2004-000991-15; Multi-centre Research Ethics Committee Ref No. 03/8/087; ClinicalTrials.gov No. NCT00135226; ISRCTN No. ISRCTN60635500.

## Protocol

Available via the ClinicalTrials.gov website.

## Declaration of competing interest

The authors declare that they have no known competing financial interests or personal relationships that could have appeared to influence the work reported in this paper.
