# Supplementary material for: Experience of linking to the NHS diabetic eye screening programme records in the ASCEND-eye randomized trial and recommendations for improvement
Source: Contemp Clin Trials Commun. 2025 Mar 28;45:101474. doi: 10.1016/j.conctc.2025.101474 (PMC11999369; doi:10.1016/j.conctc.2025.101474)
Supplement: Multimedia component 1 [file mmc1.docx]

**Supplementary Appendix 1**

This supplement contains the following items:

ASCEND Study Collaborative Group

Table S1 Summary of Applications and their Approvals by Date

## ASCEND Study Collaborative Group

**Writing Committee**

Emily Sammons (*Corresponding author)*, Louise Bowman, Marion Mafham, Jane Armitage

**Steering Committee**

*Chairman:* R Collins, *Study coordinator:* J Armitage; *Clinical coordinator:* L Bowman; *Statisticians:* S Parish, R Peto; *Administrative coordinator:* J Barton; *Lay member:* D Simpson; *Other members:* A Adler, T Aung, C Baigent, HJ Bodansky, A Farmer, R Haynes, R McPherson, M Mafham, HAW Neil, N Samani, P Sleight, P Weissberg.

**Data Monitoring Committee**

*Chair:* P Sandercock, *Members:* H Gerstein, R Gray, C Hennekens.

**Coordinating Office (Clinical Trial Service Unit, Nuffield Department of Population Health, University of Oxford):**

*Administration and support:* J Barton, L Fletcher, K Murphy (coordinators); S Hurley, R Lee, S Pickworth, M Willett, M Wincott.

*Clinical support and adjudication:* J Armitage, L Bowman, M Mafham, E Sammons

*Statistics and computing:* M Lay, S Parish; G Buck, A Murawska, W Stevens, K Wallendszus, A Young, I Hammami

*Research support:* K Melham, G Brown, J Latham-Mollart, A Brewer

**Collaborators**

*Ophthalmologists:* P Scanlon^1^, P Patel^2^

*Public Health England* (now the UK Health Security Agency): M Olson^3^

*Public Health Wales^4^:* J Kay, S Banerjee, L Evans, A Davies, M Griffiths, H Clayton

*Health Intelligence Ltd^5^:* P Kirby. M Pennington^8^, D Clarke

*Northgate Ltd^6^:* J Anslow, A Hallam, J Witts, S Egan, A Wharton

*Cheshire DESP^7^:* A Sachdev, A Derbyshire, E Williamson, K Hepplestone

*East and North Hertfordshire DESP^8^:* S Mithra, S Oliver, P Wiatrak-Olszewska

*Greater Nottingham DESP^9^:* T Gazis, K Alvey, E Wu

*Humber DESP^10^:* H Cook, N Gregory, P Parkinson

*North East London DESP^11^:* J Anderson, L Bolter

*North Nottinghamshire DESP^12^:* P Maharajan, R McFee, L Allsop, D Sowter, D Hodgson

*North Yorkshire DESP^13^:* J Thow, J Featonby, R Furnival

*Oxfordshire DESP^14^:* P Scanlon, H Lipinski, H Benjamin, T McAfee

*South East Sussex DESP^15^:* E Payne, L Still

**Funding**

The Macular Society^16^

British Heart Foundation^17^

1. Department of Ophthalmology, Gloucestershire Hospitals NHS Foundation Trust, Cheltenham General Hospital, Sandford Road, Cheltenham, GL53 7AN
2. Moorfields Eye Hospital, 162 City Road, London, EC1V 2PD
3. Health Information Consulting Ltd, Low Barn, Homestall Lane, Faversham, Kent, ME13 8UT
4. Public Health Wales Research and Evaluation Division, Knowledge Directorate, Floor 5, 2 Capital Quarter, Tyndall Street, Cardiff, CF10 4BZ
5. InHealth Intelligence, Unity House, Road 5, Winsford Industrial Estate, Winsford, Cheshire, CW7 3RB
6. Northgate Public Services (UK) Ltd, Crome Lea Business park, Madingley road, Coton, CB23 7PH
7. Cheshire Diabetic Eye Screening Programme, Eagle Bridge Health and Wellbeing Centre, Dunwoody Way, Crewe, CW1 3AW
8. East and North Hertfordshire Diabetic Eye Screening Programme, Administration Centre H4, Hertford County Hospital, North Road, Hertford, SG14 1LP
9. Greater Nottinghamshire Diabetic Eye Screening Programme, 2nd Floor, Ropewalk House, 113 The Ropewalk, Nottingham, NG1 5DU
10. Humber Diabetic Eye Screening Programme, Alderson House, Hull Royal Infirmary, Hull, HU3 2JZ
11. North East London Diabetic Eye Screening Programme, The Eye Screening Centre, Homerton University Hospital, Homerton Row, Hackney, London, E9 6SR
12. North Nottinghamshire Diabetic Eye Screening Programme, Kings Mill Hospital, Trust Admin Building - Level 2, Mansfield Road, Sutton In Ashfield, Notts, NG17 4JL
13. North Yorkshire Diabetic Eye Screening Programme, 2 Cayley Court, George Cayley Drive, Clifton Moor, York, YO30 4WH
14. Oxfordshire Diabetic Eye Screening Programme, Level 0, West Wing, John Radcliffe Hospital, Headley Way, Headington, Oxford, OX3 9DU
15. South East Sussex Diabetic Eye Screening Programme, Bexhill Hospital, East Sussex Healthcare NHS Trust, Hollier's Hill, Bexhill-on-Sea, TN40 2DZ
16. Macular Society, Crown Chambers, South Street, Andover, SP10 2BN
17. British Heart Foundation, Greater London House, 5th Floor, 180 Hampstead Road, London, UK, NW1 7AW

**Table S1 Summary of Application and their Approvals by Date**

| **Activity** | **Application Date** | **Approval Date** |
| --- | --- | --- |
| **Sponsor Agreement** | | |
| 1^st^ Substantial Amendment* | 25/08/2016 | 21/09/2016 |
| 2^nd^ Substantial Amendment^†^ | 02/11/2016 | 17/11/2016 |
| 3^rd^ Substantial Amendment^‡^ | 27/12/2018 | 03/01/2019 |
| 4^th^ Substantial Amendment^§^ | 29/10/2019 | 31/10/2019 |
| **Invitations to collaborate sent to the clinical leads (CL) and programme managers (PM) of NHS Diabetic Eye Screening Programmes (DESPs) in England and Wales** | | |
| Collaborations established with CLs and PMs | January 2017 | - |
| **Diabetic Eye Screening Research Advisory Committee Approvals** | | |
| Public Health England (annual meeting on 13^th^ June) | 14/05/2018 | 21/07/2018 |
| Public Health Wales | 27/11/2018 | 08/08/2019 |
| **Multi-Centre Research Ethics Committee and Health Research Authority^¶^ Approval** | | |
| 1^st^ Substantial Amendment* | 22/09/2016 | 19/10/2016 |
| 2^nd^ Substantial Amendment^†^ | 18/11/2016 | 14/12/2016 |
| 3^rd^ Substantial Amendment^‡^ | 04/01/2019 | 28/01/2019 |
| 4^th^ Substantial Amendment^§^ | 31/10/2019 | 01/11/2019 |
| **Collaborating DESP Research and Development (R&D) Approvals** | | |
| Cheshire DESP | 10/01/2018 | 15/07/2019 |
| East and North Hertfordshire DESP | 29/01/2019 | 11/06/2019 |
| Greater Nottingham DESP | 27/11/2018 | 02/07/2019 |
| Humber DESP | 27/11/2018 | 31/10/2019 |
| North East London DESP | 27/11/2018 | 07/01/2019 |
| North Nottinghamshire DESP | 28/01/2019 | 27/06/2019 |
| North Yorkshire DESP | 27/11/2018 | 26/09/2019 |
| Oxfordshire DESP | 27/11/2018 | 10/06/2019 |
| South East Sussex DESP | 29/01/2019 | 14/06/2019 |
| Northgate-controlled DESPs | 20/06/2019 | 27/01/2020 |
| Health Intelligence DESPs | 12/02/2020 | 23/08/2021 |
| Diabetic Eye Screening Wales | 27/11/2018 | 08/08/2019 |
| **Other Approvals** | | |
| **Data Protection Impact Assessment** | 05/03/2020 | 11/10/2020 |
| **Third-Party Security Assessments** (Northgate and Health Intelligence Ltd) | 18/05/2020 | 03/06/2020 |
| **Financial Services Contract (Northgate only**)** | 20/06/2019 | 27/01/2020 |
| **Data Sharing Agreements (simultaneously sought with R&D approvals)** | | |
| Cheshire DESP | - | 15/07/2019 |
| East and North Hertfordshire DESP | - | 24/06/2019 |
| Greater Nottingham DESP | - | 02/07/2019 |
| Humber DESP | - | 10/10/2019 |
| North East London DESP | - | 07/01/2019 |
| North Nottinghamshire DESP | - | 27/06/2019 |
| North Yorkshire DESP | - | 26/09/2019 |
| Oxfordshire DESP | - | 10/06/2019 |
| South East Sussex DESP | - | 14/06/2019 |
| Northgate-controlled DESPs | - | 27/01/2020 |
| Health Intelligence DESPs | - | 23/08/2021 |
| Diabetic Eye Screening Wales | - | 08/08/2019 |

*To approve a sub-study protocol and participant-facing materials.

^†^To approve an extension to the ASCEND trial ethics approval to 2037, permitting further follow-up and sub-studies, such as ASCEND-Eye

^‡^To approve the addition of NHS Diabetic Eye Screening Programmes as research sites (relevant to areas that were not included as Participant Identification Centres in ASCEND).

^§^To approve the integration of ASCEND-Eye and its documentation into the ASCEND trial protocol

^¶^HRA approval only became necessary after the 3rd substantial amendment was approved.

**Health Intelligence Ltd provided their collaboration for free
